# Progressing Insights into the Role of Dietary Fats in the Prevention of Cardiovascular Disease

**DOI:** 10.1007/s11886-016-0793-y

**Published:** 2016-09-20

**Authors:** Peter L. Zock, Wendy A. M. Blom, Joyce A. Nettleton, Gerard Hornstra

**Affiliations:** 1 Unilever R&D Vlaardingen, Olivier van Noortlaan 120, 3133 AT Vlaardingen, The Netherlands; 2 Member of the IUNS-associated International Expert Movement to Improve Dietary Fat Quality, ScienceVoice Consulting, 2931 Race Street, Denver, CO 80205 USA; 3 Member of the IUNS-associated International Expert Movement to Improve Dietary Fat Quality, Maastricht University, Brikkenoven 14, 6247 BG Gronsveld, Netherlands

**Keywords:** Review, Saturated fat, Unsaturated fat, Omega-6, Omega-3, Blood lipids and lipoproteins

## Abstract

Dietary fats have important effects on the risk of cardiovascular disease (CVD). Abundant evidence shows that partial replacement of saturated fatty acids (SAFA) with unsaturated fatty acids improves the blood lipid and lipoprotein profile and reduces the risk of coronary heart disease (CHD). Low-fat diets high in refined carbohydrates and sugar are not effective. Very long-chain polyunsaturated n-3 or omega-3 fatty acids (n-3 VLCPUFA) present in fish have multiple beneficial metabolic effects, and regular intake of fatty fish is associated with lower risks of fatal CHD and stroke. Food-based guidelines on dietary fats recommend limiting the consumption of animal fats high in SAFA, using vegetable oils high in monounsaturated (MUFA) and polyunsaturated fatty acids (PUFA), and eating fatty fish. These recommendations are part of a healthy eating pattern that also includes ample intake of plant-based foods rich in fiber and limited sugar and salt.

## Introduction

Dietary fats are important in the prevention of CVD, as documented in many studies over the past 60 years. Early research in the 1950s and 1960s focused on saturated fatty acids (SAFA) and vegetable monounsaturated (MUFA) and polyunsaturated fatty acids (PUFA) in relation to blood cholesterol levels and the risk of coronary heart disease (CHD). Later studies revealed the importance of very long-chain (≥20 carbons) omega-3 PUFA (n-3 VLCPUFA) present in fish and seafood and the adverse effects of trans-fatty acids (TFA) [1, 2]. Most recently, research on dietary fat and the prevention of metabolic diseases has focused on whole dietary patterns, such as the Mediterranean diet, and specific food sources of fatty acids.

Thus, extensive evidence underpins current dietary recommendations and food-based guidelines to reduce SAFA intake and replace them with unsaturated fatty acids [3]. However, several recent publications have questioned the evidence and rationale for dietary fat recommendations, especially for SAFA and omega-6 (n-6) PUFA (mainly linoleic acid, LA) intakes [4, 5]. These publications and the media attention they attracted have created confusion among consumers and health care professionals about the health effects of different dietary fats.

This narrative review provides an overview of recent analyses and selected studies on dietary fatty acids and the risk of CVD in humans. Most of the scientific literature describes the effects of dietary fat consumption on blood lipids, lipoproteins, and risk of CHD, although many studies reported total CVD as the primary endpoint. There is also an extensive literature on the effects of dietary fats on other CVD risk factors and endpoints. We describe human studies with research designs of the highest relevance for evaluating the effects of diet on human health and setting guidelines [6]. These include long-term randomized controlled trials on clinical endpoints (RCTs), prospective analyses from large population studies, and controlled dietary intervention studies on established markers of cardiometabolic risk (metabolic trials).

Low-density lipoprotein cholesterol (LDL-C) and blood pressure (BP) are established causal risk factors for CHD and stroke, respectively, and therefore a primary treatment target for the prevention of CVD [7–9]. Low high-density lipoprotein cholesterol (HDL-C) levels, a high total cholesterol (TC)/HDL-C ratio, and elevated triglyceride (TG) concentrations are seen as independent predictors of cardiovascular risk [10–12]. Dietary fatty acids may also affect risk via markers of endothelial and cardiac function, chronic inflammation, and blood clotting tendency [13]. Diabetes confers a 2-fold risk for CHD and is a major risk factor that is modifiable by diet and lifestyle [14]. This review therefore also considers new insights in the effects of dietary fatty acids on markers of insulin sensitivity and risk of type II diabetes mellitus (T2DM).

## Dietary Fatty Acids

Dietary fats consist predominantly of triacylglycerol or triglycerides, formed by fatty acids esterified to glycerol. Fatty acids can differ in chain length and the number of double bonds, location of the double bond in the carbon chain (e.g., n-6, n-3), and the configuration of the double bond (*cis* or *trans*). Most fat calories (energy) come from SAFA with 12–18 carbon atoms (C12:0-C18:0, no double bond), MUFA (mostly *cis*-C18:1 oleic acid), and PUFA. PUFA, mainly present in seed oils, are predominantly n-6 (omega-6) linoleic acid (C18:2), and some n-3 alpha-linolenic acid (C18:3, ALA). Smaller amounts of n-3 PUFA (milligrams instead of grams) occur in seafood as very long-chain fatty acids with 5 or more double bonds (predominantly C20:5 eicosapentaenoic acid [EPA] and C22:6 docosahexaenoic acid [DHA]). Trans fatty acids (TFA) are unsaturated fatty acids with one or more double bonds in the *trans* configuration.

Table 1 shows that all common edible fats and fat-rich foods contain SAFA, MUFA, and PUFA, but in different amounts. Animal products are relatively rich in SAFA, while vegetable oils and nuts are typically rich in PUFA. MUFA occur in substantial amounts in both animal and vegetable products. Although some vegetable fats, such as coconut and palm kernel oil, are very high in SAFA, meat and dairy are the main food sources of SAFA in western diets. Most dietary PUFA come from vegetable oils, but also some from cereals, fish, and meat [15–17].

**Table 1:** Fatty acid composition of common foods and fats

Gram/100 g product	Total SAFA	*cis*-MUFA	*cis*-PUFA	Total fat
Butter	50.5	20.4	3.0	81.1
Whole milk	1.9	0.8	0.2	3.3
Reduced fat (2 %) milk	1.2	0.1	0.0	2.0
Whole milk yogurt	2.1	0.9	0.1	3.3
Cheese, cheddar	18.9	8.3	1.4	33.3
Cheese, Edam	17.6	8.1	0.7	27.8
Salmon, Atlantic, wild, raw	1.0	2.1	2.5	6.3
Salmon, Atlantic, farmed, raw	3.1	3.8	3.9	13.4
Ground beef (17 % fat)	6.8	7.4	0.7	17.1
Pork, cured ham	1.0	1.9	0.6	5.1
Lamb, ground, raw	10.2	9.6	1.9	23.4
Cashews, roasted	9.2	27.3	7.8	46.4
Peanuts, roasted	7.7	26.2	9.8	49.7
Walnuts	6.1	8.9	47.2	65.2
Almonds	3.8	31.5	12.3	49.9
Avocado	2.1	9.8	1.8	15.4
Cocoa butter oil	59.7	32.9	3.0	100.0
Palm oil	49.3	37.0	9.3	100.0
Palm kernel oil	81.5	11.4	1.6	100.0
Coconut oil	82.5	6.3	1.7	99.1
Soybean oil	15.3	22.7	56.7	100.0
Sunflower oil	10.3	16.5	65.7	100.0
Canola (rapeseed) oil	7.4	63.2	27.8	100.0
Olive oil	13.8	73.0	10.5	100.0
Fish oil, sardine	29.9	33.8	31.9	100.0
Fish oil, cod liver	22.6	46.7	22.5	100.0

Source: USDA National Nutrient Database for Standard Reference, release 28 [16]

Although the intake of SAFA in western societies has substantially deceased in the past decades [18–20], analyses of global intake data indicate that a majority of the population still consumes more SAFA and less PUFA than recommended for the prevention of chronic disease [3]. Since the discovery of their adverse effects on CHD risk [21], TFA from partially hydrogenated oils have been removed from many foods, resulting in substantially decreased intakes. Nowadays, total TFA intakes in most populations are not higher than 1 % of energy, and contributions of TFA from ruminant sources (dairy, beef) are often larger than from partially hydrogenated oils [22–25].

## Effects of the Major Classes of Fatty Acids (SAFA, MUFA, PUFA) on Cardiovascular Risk

Most studies on dietary fats and risk of CVD investigated the effects of the main fatty acid classes SAFA, MUFA, and PUFA on the blood lipoprotein profile and their relation with CHD risk. Because fatty acids deliver a significant amount of daily energy, there is—unlike for studies with drugs—no appropriate placebo to investigate their independent effects. Therefore, the effects of SAFA, MUFA, and PUFA should be expressed relative to a similar amount of energy from the other macronutrients that replace the fatty acid under study, e.g., other fatty acids, carbohydrates, or protein. Specifying the replacement nutrient is crucial, because it affects the comparison of, for example, SAFA with other fatty acids or with carbohydrates. Failure to specify the nutrient of comparison is a major source of confusion about the health effects of dietary fats.

### Effects on Blood Lipids and the Lipoprotein Risk Profile

The effects of fatty acids on plasma LDL-C, HDL-C, and TG levels have been established by many strictly controlled dietary intervention studies [26, 27••]. Replacement of SAFA with PUFA not only lowers LDL-C, but also the TC/HDL-C ratio. Replacement with MUFA has similar, but smaller, effects on blood lipids [27••]. Replacement of SAFA with carbohydrates, i.e., a low-fat diet, lowers LDL-C but also HDL-C and has no effect on the TC/HDL-C ratio. High-carbohydrate, low-fat diets also raise fasting blood TG levels compared with SAFA, MUFA, and PUFA. Thus, reducing dietary SAFA and replacing it with carbohydrates, i.e., a low-fat diet, do not improve the overall blood lipid and lipoprotein risk profile. TFA have adverse effects on the blood lipid profile compared with all other fatty acids and carbohydrates [1, 26]. The effects of protein on blood lipids are less extensively studied. The available metabolic trials suggest that protein, like MUFA and PUFA, reduces the TC/HDL-C ratio and TG levels compared with carbohydrates [28, 29].

### Meta-analyses of Data on Fatty Acid Replacement and Clinical CVD Endpoints

The evidence from studies on clinical endpoints (Table 2) is consistent with the effects of fatty acids on the blood lipid and lipoprotein risk profiles [26, 27••]. Table 2 illustrates that significant reductions in CHD risk are observed when SAFA is compared with or replaced by PUFA, but changes in risk are less clear when the replacement nutrient is not specified or when SAFA is compared with carbohydrates.

**Table 2:** Recent meta-analyses of prospective cohort studies and randomized clinical endpoint trials on the relation between fatty acid intake and risk of coronary heart disease

Observational studies		
Author	Macronutrients replacing the calories from SAFA or PUFA specified	
	YES (substitution models used)	NO
Jakobsen 2009, AJCN [34]	5%E replacement SAFA by PUFA -RR: 0.87^a^ (0.77–0.97) CHD events -RR: 0.74^a^ (0.61–0.89) CHD deaths 5%E replacement SAFA by MUFA -RR: 1.19^a^ (1.00–1.42) CHD events -RR: 1.01 (0.73–1.41) CHD deaths 5%E replacement SAFA by carbs -RR: 1.07^a^ (1.01–1.14) CHD events -RR: 0.96 (0.82–1.13) CHD deaths	
Skeaff & Miller, 2009, Ann Nutr Metab [30]		High versus low SAFA intake -RR: 0.93 (0.83–1.05) CHD events -RR: 1.14 (0.82–1.60) CHD deaths High versus low MUFA intake -RR: 0.87 (0.74–1.03) CHD events -RR: 0.85 (0.60–1.20) CHD deaths High versus low PUFA intake -RR: 0.97 (0.74–1.27) CHD events -RR: 1.25^a^ (1.06–1.47) CHD deaths High versus low VLCPUFA intake -RR: 0.87 (0.71–1.10) CHD events -RR: 0.82^a^ (0.71–0.94) CHD deaths
Siri-Tarino 2010, AJCN [31]	Replacement SAFA by PUFA -RR: 0.93 (0.80–1.10) total CHD Replacement SAFA by carbs -RR: 1.02 (0.88–1.16) total CHD	High versus low SAFA intake -RR: 1.07 (0.96–1.19) total CHD
Farvid 2014, Circulation [35•]	5%E replacement SAFA by LA -RR: 0.91^a^ (0.86–0.96) CHD events -RR: 0.87^a^ (0.82–0.94) CHD deaths	High versus low LA intake -RR: 0.85^a^ (0.78–0.92) CHD events -RR: 0.79^a^ (0.71–0.89) CHD death
Chowdhury 2014, Ann Intern Med [32]		High versus low SAFA intake -RR: 1.03 (0.98–1.07) total CHD High versus low MUFA intake -RR:1.00 (0.91–1.10) total CHD High vs low PUFA intake -ALA: RR:0.99 (0.86–1.14) total CHD -VLCPUFA: RR:0.87^a^ (0.78–0.97) total CHD -LA: RR:0.98 (0.90–1.06) total CHD
De Souza 2015, BMJ [33]		High versus low SAFA intake -RR: 1.15 (0.97–1.36) CHD death -RR: 1.06 (0.95–1.17) total CHD
Pan 2012, AJCN [79]		High versus low ALA intake -RR: 0.94 (0.85–1.04) total CHD -RR: 0.80^a^ (0.65–0.98) fatal CHD -RR: 0.84 (0.61–1.15) nonfatal CHD
Vedtofte 2014, Br J Nutr [45]		each additional g/day intake of ALA -RR: 0.88 (0.75–1.02) CHD events -RR: 0.88 (0.68–1.14) CHD death
Randomized controlled trials		
Author	Energy substitution models used?	
	YES	NO
Skeaff and Miller 2009, Ann Nutr Metab [30]		PUFA-SAFA modified trials -RR: 0.83^a^ (0.69–1.00) CHD events -RR: 0.84 (0.62–1.12) CHD death Fish or omega-3 VLCPUFA trials -RR: 0.88 (0.76–1.01) CHD death -RR: 0.89^a^ (0.82–0.98) CHD event
Ramsden 2010, BJN [121]		High PUFA/Low SAFA diet vs control diet ^b^ -RR: 0.86 (0.68–1.07) non-fatal MI -RR: 0.91 (0.74–1.10) CHD death
Mozaffarian 2010, PLoS Med [42]	5%E replacement SAFA by PUFA RR: 0.90^a^ (0.83–0.97) total CHD	High PUFA/low SAFA diet vs control diet -RR: 0.81^a^ (0.70–0.95) CHD events
Schwingshackl 2014, BMJ Open [122]		High PUFA/low SAFA diet vs control diet -RR: 0.91 (0.65–1.29) total MI
Harcombe 2015^#^, Open Heart [4]		High PUFA/low SAFA diet vs control diet ^b^ -RR: 0.99 (0.78–1.20) CHD death
Hooper 2015, The Cochrane Library [43••]	Replacement of SAFA by PUFA -RR: 0.80 (0.63–1.03) non-fatal MI -RR: 0.83 (0.67–1.02) total MI -RR: 0.76^a^ (0.57–1.0) CHD events - RR: 0.98 (0.74–1.28) CHD death	Low SAFA diet vs control diet ^b^ -RR:0.95 (0.80–1.13) non-fatal MI -RR: 0.90 (0.80–1.01) total MI -RR 0.87 (0.74–1.03) CHD events -RR 0.98 (0.84–1.15) CHD death
Chowdhury 2014, Ann Intern Med [32]		PUFA diet vs control diet^b^ -ALA: RR: 0.97 (0.69–1.36) total CHD -VLCPUFA: RR: 0.94 (0.86–1.03) total CHD -LA: RR: 0.86 (0.69–1.07) total CHD

Note that different meta-analyses are based on largely the same underlying individual prospective cohort studies and RCTs

*ALA* alpha-linoleic acid, *carbs* carbohydrates, *CHD* coronary heart disease, *LA* linoleic acid, *MI* myocardial infarction, *MUFA* monounsaturated fatty acids, *PUFA* polyunsaturated fatty acids, *RR* relative risk, *SAFA* saturated fatty acids, *VLCPUFA* very long-chain polyunsaturated fatty acids

^a^Statistically significant relative risks

^b^Mix of high PUFA and low-fat diets

#### Meta-analyses of Observational Studies

Meta-analyses of prospective cohort studies that investigated SAFA consumption without taking the replacement nutrient into account found no statistically significant associations between risk of CHD and SAFA intakes [30–33]. On the other hand, the meta-analyses that did model the replacement nutrient (Table 2, middle column) showed significantly lower CHD risks when PUFA partially replaced SAFA calories, but not when carbohydrate replaced SAFA (low-fat diets) [31, 34, 35•]. Jakobsen et al. [34] found no relationship when MUFA was substituted for SAFA (Table 2).

It is not surprising that differences in SAFA intake as such do not show significant associations with CHD risk. When the replacement nutrient is not specified, energy from SAFA is conceptually compared with the average rest of the diet. In practice, this is mainly energy from carbohydrates, which do not benefit CHD risk [26, 27••]. There are also several methodological reasons why observational studies do not find a relation between SAFA intake and CHD risk, as discussed in detail by others [36].

#### Recent Prospective Studies on SAFA, MUFA, PUFA, Carbohydrates, and CVD

More recent prospective cohort studies confirm that replacement of SAFA with PUFA is associated with a lower risk of CVD endpoints, in particular CHD [37••, 38, 39•, 40], although not necessarily in all studies [41]. An analysis in 125,000 US men and women reported that lower SAFA intakes and higher PUFA intakes, especially LA, were associated with lower total mortality, including CVD, cancer, and neurodegenerative disease [40].

Another recent analysis of the same cohorts showed that not only PUFA, but also MUFA and whole-grain carbohydrates are beneficial replacements for SAFA [37••]. In addition, high PUFA intakes were associated with a lower CHD risk as compared with refined carbohydrates and sugar. This finding suggests that PUFA have beneficial effects on CHD risk independent of SAFA substitution.

#### Meta-analyses of RCTs

The results of RCTs are in line with observations from cohort studies. The meta-analyses of RCTs that compared dietary SAFA with PUFA reported significant reductions in CHD events [42, 43••]. This was less clear for analyses of RCTs that did not consider the replacement nutrient (Table 2). There are no long-term RCTs that specifically studied replacement of SAFA by MUFA, except in combination with other dietary changes (for example, PREDIMED and the Lyon diet heart study [38, 44]). There are also no RCTs on TFA. In contrast to *cis*-MUFA, TFA have adverse effects on blood lipid and lipoprotein profiles [1, 26, 27••], and high intakes of TFA are consistently associated with higher CHD risk in prospective cohort studies [33].

It should be noted that the different estimates presented in Table 2 are based on largely the same underlying data, although the various meta-analyses differed in inclusion or exclusion criteria and in the weighting of the individual studies. The most comprehensive systematic review and meta-analysis on SAFA reduction and risk of CVD by Hooper et al. [43••], included the data from 15 RCTs. It concluded that lowering the intake of SAFA by replacing it with mixed carbohydrates or PUFA reduces the risk of combined cardiovascular events by an average of 17 % (*P* = 0.01). This effect was mainly due to the studies that replaced SAFA by PUFA, with a significant risk reduction of 24 % for CHD events (Table 2). SAFA reduction by using fat-reduced diets did not change cardiovascular risk. No statistically significant effect on cardiovascular and all-cause mortality or stroke was observed [43••].

Overall, the available data from metabolic trials, prospective cohorts, and RCTs provide strong evidence that replacement of SAFA with PUFA lowers CHD risk. In addition, PUFA consumption in itself is associated with reduced CHD risk, even if the replaced nutrients are not taken into account [37••, 45]. The combined data indicate that a 5%E replacement of SAFA with PUFA reduces the risk of CHD by about 10 % (Table 2 [42]).

Replacement of SAFA with carbohydrates, i.e., low-fat diets, does not confer cardiovascular benefit (Table 2). This finding is supported by the Women’s Health Initiative study of Howard et al. [46], which found that behavioral intervention aimed at reducing total fat intake to 20 % of calories had no significant effect on the incidence of CHD, stroke, or overall CVD.

Replacement with MUFA has favorable effects on blood lipids, but data on CHD endpoints are too limited to draw conclusions.

### Effects of Replacing Dietary Fatty Acids on Other, Non-lipid CVD/CHD Markers

#### Effects on BP and Endothelial Function

Replacing calories from different fatty acids for each other or carbohydrates had no or only small effects on BP in several older metabolic trials [47–49]. The recent Omniheart trial investigated the effects on both serum lipids and BP of 6-week interventions with reduced SAFA diets rich in either protein, MUFA, or carbohydrates [28]. Compared with carbohydrates, the MUFA diet decreased BP by 1–3 mmHg, but only in subjects with hypertension [28]. The recent DIVAS study [50] investigated the effects of SAFA, MUFA, and PUFA on several CVD risk factors in a 16-week intervention study in 202 men and women. Replacement of SAFA with MUFA or PUFA resulted in favorable effects on BP (night systolic BP) and E-selectin (a pro-inflammatory cytokine, associated with hypertension) but had no significant effect on vascular function as measured by flow-mediated dilatation. These studies suggest that exchanging the major classes of dietary fatty acids for each other may have only a small effect on BP.

#### Effects on Glucose and Insulin Metabolism and Risk of T2DM

Several metabolic trials reported that exchanging the major classes of dietary fatty acids affects glucose-insulin homeostasis. Other reviews concluded that replacing SAFA with MUFA or PUFA probably increases insulin sensitivity, although the data were not conclusive [51, 52]. The favorable effects of MUFA as replacement for SAFA on insulin sensitivity found in the KANWU trial [53] have not been confirmed by other studies [54, 55]. Other metabolic trials found that iso-calorically replacing SAFA with PUFA improved insulin sensitivity [56] and reduced hepatic fat disposition [57]. The LIPOGAIN trial studied the effects of a 7-week hypercaloric diet (=overfeeding) with SAFA or n-6 PUFA on liver fat and body composition in 40 subjects. The PUFA group gained significantly less hepatic, visceral, and total fat compared with the SAFA group [58].

Imamura et al. [59] performed a meta-analysis of 102 controlled metabolic trials on dietary fatty acids and reported effects on glucose and insulin metabolism. Substituting 5%E PUFA for SAFA decreased fasting glucose by 0.04 mmol/L. PUFA also decreased fasting insulin by −1.6 pmol as compared with carbohydrates, but effects of replacement with MUFA on glucose and insulin were less clear. Subgroup analysis showed that both MUFA and PUFA significantly decreased HbA1c and HOMA-IR (markers of insulin resistance) when replacing either 5%E carbohydrates or SAFA [59]. The authors estimated that these effects translate into a 22 % reduction in diabetes risk and a 7 % reduction in CVD risk [59].

#### Recent Observational Studies on SAFA, MUFA, PUFA, and risk of T2DM

In a meta-analysis of six prospective cohort studies, intakes of SAFA were not related to diabetes risk when the substitution nutrients were not considered [60]. This was also observed in an Australian cohort, not included in this meta-analysis [61]. However, in an analysis of US women, substitution of 3%E from PUFA for SAFA was associated with a 16 % lower risk of T2DM [62]. High intake of MUFA was not clearly associated with lower risk of T2DM in these and other prospective studies [62–64].

Blood fatty acids that cannot be or are hardly synthesized by the human body, such as n-6 and n-3 PUFA, can be used as biomarkers of their dietary intakes. Yary et al. reported an inverse association between plasma total n-6 fatty acids, LA, as well as longer chain arachidonic acid, and the risk of diabetes in a Finnish population [65]. This is in line with the observations from several other cohort studies on plasma LA and T2DM endpoints [61, 66], but not all [67]. The largest prospective study on blood PUFA and T2DM risk, to date, is a pooled analysis of EPIC InterAct [68]. This analysis showed inverse associations of risk for both circulating LA and ALA. T2DM risk was 20 % lower per SD increase in LA and 7 % lower per SD increase in ALA [68].

Recent metabolic trials and cohort studies provide strong evidence that dietary unsaturated fatty acids, in particular PUFA, favorably affect glucose and insulin metabolism and reduce the risk of T2DM. More long-term studies should further substantiate the potential of dietary fatty acid composition to prevent T2DM and, in this way, reduce the risk of CHD and other cardiovascular complications of diabetes. Regardless, diabetes patients are at high risk of CHD. Therefore, replacement of SAFA by PUFA is important to improve their blood lipid and lipoprotein risk profile.

## Specific SAFA and PUFA and Cardiometabolic Risk

### Differences Between Specific SAFA and Food Sources

Dietary SAFA of different chain length, mainly C12-C18, differ in their effects on blood lipids and lipoproteins. Compared with carbohydrates, lauric acid (C12), myristic acid (C14), and palmitic acid (C16) increase LDL-C, whereas stearic acid (C18) does not [26, 27••]. However, C18 also does not increase HDL-C (whereas C12-C16 SAFA do) and has the same effects on the TC/HDL-C ratio as carbohydrates.

There is only scarce and inconsistent evidence from prospective cohorts and none from RCTs on the relationship between different dietary SAFA and CHD endpoints [41, 69]. Moreover, it is difficult to disentangle the relations of specific SAFA with risk in observational studies, because different SAFA, in particular C16 and C18, come for a large part from the same foods [15, 16, 23, 41], and their intakes are highly correlated [17, 41]. In addition, the contributions of C12 and C14 (and shorter chain fatty acids) to energy intake are limited, making their associations with disease endpoints in observational studies uncertain [41]. Thus, it is unclear whether the differential effects of specific SAFA on blood lipids translate into differences in the risk of CHD events.

One prospective study, the Multi-Ethnic Study of Atherosclerosis (MESA) study investigated different foods in relation to SAFA intake and CHD risk. Each 5%E increase in dairy SAFA intake was associated with a 38 % lower risk of CVD (HR: 0.62 [0.47–0.82]), while 5%E from meat SAFA was associated with a 48 % higher risk (HR: 1.48 [0.98–2.23]) [70], suggesting that the food source of SAFA may modulate the effects of SAFA with CVD risk.

A meta-analysis of five metabolic trials comparing the effects of hard cheese with butter indicated that SAFA from cheese may increase LDL-C and HDL-C levels less than SAFA from butter [71]. However, a recent metabolic trial [72] found that the effects on blood lipids of SAFA from hard cheese were not significantly different from SAFA from meat, suggesting that the cheese matrix does not influence the effects of SAFA on blood lipids. Further studies should sort out the discrepancies among food sources of SAFA, in particular dairy, and their effects on cardiometabolic risk. It should be noted that butter, which is mostly SAFA and raises LDL-C [26], is generally *not* included with the dairy foods in observational studies.

**Fig. 1:**
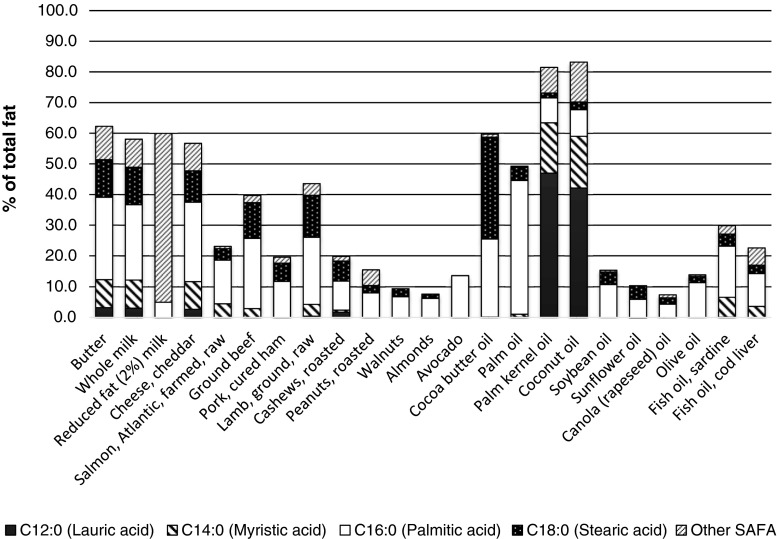
Specific saturated fatty acid composition of common foods and fats

Popular belief holds that coconut oil is healthy, a notion not supported by scientific data [73, 74]. Coconut oil is an edible oil with a very high total SAFA content (80 %). A common misconception is that the SAFA in coconut oil are mainly medium chain fatty acids, which are metabolized differently from long-chain SAFA. Actually, coconut oil is mainly C12:0 lauric acid and C14:0 myristic acid (Fig. 1), which have potent LDL-C-raising effects [26, 27••]. Coconut oil should therefore not be advised for people who should or want to reduce their risk of CHD.

### N-6 and N-3 PUFA

LA is the predominant n-6 PUFA in food fats (Table 1), accounting for about 80–90%E [22] of total dietary PUFA. The main dietary n-3 fatty acid is ALA [22, 75]. N-3 VLCPUFA have a variety of cardioprotective effects [13, 76], but their amounts in the diet are very small and cannot substantially replace calories from other nutrients. Therefore, the evidence about the beneficial effects of PUFA as isocaloric fatty acid replacement for SAFA on CHD endpoints (Table 2) is largely based on vegetable PUFA, being mainly LA and some ALA. Because LA and ALA come largely from the same vegetable oils and foods, and LA intake is much higher than ALA, it is difficult to separate their effects in RCTs and observational studies (Table 2).

The effects of ALA on blood lipids are therefore less established than those of LA [26, 27••]. The evidence from metabolic trials that specifically investigated ALA indicates that LA and ALA have per gram similar effects on the blood lipid and lipoprotein profile [77, 78]. Meta-analyses of prospective observational studies report lower risks of CVD/CHD with higher intakes of both LA and ALA. The meta-analysis of prospective studies by Farvid (Table 2) found a 15 % lower risk of CHD events and a 21 % lower risk of CHD deaths for the highest vs the lowest LA intakes, without taking the replaced nutrient into account. An increment of 5%E as LA modeled at the expense of SAFA was associated with a 10 % lower risk of CHD events and a 13 % lower risk of CHD deaths [35•]. In a recent pooled analysis of individual data from eight large cohort studies, not taking nutrient replacement into consideration, each gram of additional ALA (∼0.5%E), was associated with non-significant 15 and 23 % lower risks of CHD events and CHD deaths, respectively, in men. Results for women were inconsistent [45].

Another meta-analysis of prospective studies on circulating ALA and CVD found a 10 % lower CVD risk for the highest vs the lowest ALA intakes (replaced nutrients not taken into account) [79]. The investigators also reported a non-significant 20 % lower risk for participants in the upper vs the lower tertile of ALA levels in blood or tissue. A recent cohort study not included in this meta-analysis did not find significant associations between ALA intake and risk of myocardial infarction [80].

Two recent prospective studies observed that CHD endpoints were inversely associated with higher plasma LA [81] and ALA levels [82]. However, not all studies reported a relation between blood ALA or LA and incidence of CVD [83]. A large analysis that pooled data from 19 cohort studies worldwide reported that a 1-SD increase in blood ALA as well as in n-3 VLCPUFA was associated with a 10 % lower risk of fatal CHD. Associations with non-fatal or total CHD were not evident [84]. Preliminary analyses of n-6 fatty acids in this dataset indicate that higher levels of LA in blood and tissue were also associated with lower CHD risk [85].

The RCTs that demonstrated effects of replacing SAFA with vegetable PUFA on CHD endpoints applied intervention diets in which PUFA was predominantly LA (80–95 %) (Table 2) [35•, 42]. There are no recent RCTs that exclusively studied LA on RCTs. Meta-analyses of older data by Ramsden et al. [5, 86] suggested that a specific ALA content of the diet next to LA can be important for cardiovascular outcome. However, these analyses are based on a limited number of RCTs [86] of which some had serious limitations, such as short duration and inadequate compliance.

Only a few RCTs directly investigated the effects of ALA as linseed (flaxseed) oil supplementation on CVD endpoints, and no RCTs directly compared ALA with SAFA. Linseed oil supplementation (10 g/day) had no significantly different effects compared with LA-rich oils in early studies [87, 88]. The most recent RCT reported a non-significant lower risk of cardiovascular events with 2 g/day supplemental ALA replacing oleic acid in margarine [89]. The meta-analysis of Chowdhury of RCTs on diets supplemented with different PUFA [32] did not find significant effects, for either ALA or LA-rich diets (Table 2) (RR: 0.97 [0.69–1.36] and RR: 0.86 [0.69–1.07], respectively). Taken together, the data indicate that intakes of both LA and ALA are related to a lower risk of CVD. The evidence is particularly strong for vegetable PUFA combined (mainly LA and some ALA).

N-3 VLCPUFA from fish and other seafood, mainly EPA and DHA, constitute only a fraction (∼1 %) of total dietary PUFA. However, on a gram per gram basis, n-3 VLCPUFA are more bioactive than the bulk of other dietary PUFA [90], and they affect several cardiovascular processes and risk markers [13, 76]. The effect of supplemental intakes of n-3 VLCPUFA (g/day) on plasma TG levels are well established. N-3 VLCPUFA dose-dependently lower plasma TG levels up to ∼25 % [91]. High doses of n-3 VLCPUFA also modestly lower BP by 1–2 mmHg [92] and heart rate by 1–2 beats per minute [93]. N-3 VLCPUFA inhibit pro-inflammatory processes [94, 95] and may affect thrombosis [96]. Anti-arrhythmic actions of n-3 VLCPUFA are supported by in vitro and animal experiments [97], although effects on the prevention of fatal arrhythmias are not confirmed by human studies on RCTs [13, 98, 99]. Unlike vegetable PUFA, n-3 VLCPUFA do not lower LDL-C [100]. In contrast to n-6 LA, n-3 VLCPUFA probably do not affect insulin sensitivity and diabetes risk [51].

Evidence for the primary prevention of CVD by n-3 VLCPUFA is largely based on observational studies in the general population. Many prospective cohort studies show lower risks of fatal CHD and stroke with higher consumption of (fatty) fish and seafood in the general population [101, 102]. In addition, a recent pooled analysis of 19 cohort studies reported that higher plasma concentrations of DHA and its precursors DPA and ALA (but not EPA) were associated with a modest but significantly lower incidence of fatal CHD [84].

Evidence for the efficacy of n-3 VLCPUFA in the secondary prevention of CVD is mainly based on RCTs with n-3 VLCPUFA as supplements. While earlier RCTs demonstrated a benefit [30], more recent RCTs in medically treated patients with CVD do not confirm that 600–1000 EPA + DHA mg/day as purified fish oil lowers the risk of a secondary CHD event [103]. These results have raised questions about the clinical relevance of supplemental EPA + DHA intake in such patients. However, there may be methodological reasons why recent RCTs did not find significant effects. The trials were performed in patients on intensive drug treatment [103, 104], some had a relative short intervention periods [89, 104, 105], or only a modest dose of n-3 VLCPUFA [89]. The lack of effects in recent RCTs therefore does not disprove that marine n-3 fatty acids have beneficial effects on CVD risk in the general population, and food-based dietary guidelines have not changed advice to eat fatty fish or to consume 250 mg EPA + DHA per day. Several large RCTs on CVD endpoints (REDUCE-IT [NCT01492361], STRENGTH [NCT02104817], ASCEND [NCT00135226], VITAL [NCT01169259]) in progress should provide more conclusive answers about the efficacy of EPA + DHA supplements in the prevention of cardiovascular events.

## Recommendations and Food-Based Guidelines on Dietary Fats

There is general consensus that the fatty acid composition of dietary fat, rather than its total amount is the most important feature for reducing cardiovascular risk. Most dietary recommendations for the general population agree that SAFA should be partially replaced by unsaturated fatty acids, in particular vegetable PUFA, that TFA intakes should be avoided, and that the diet should contain n-3 VLCPUFA [106–111].

Most dietary recommendations advise limiting SAFA intake to 10%E and consuming 6–11%E as PUFA. MUFA constitutes the remainder of total dietary fat intake, for which current recommendations are more liberal than 30 years ago. FAO/WHO recommends 20–35%E, and the recent dietary guidelines for Americans do not set an upper limit for total fat intake anymore [3, 106].

Some health professional organizations have issued dietary guidelines for the reduction of CVD risk and LDL-C levels, recommending the consumption of <7%E from SAFA [112–114]. Although such low SAFA intakes are associated with lower TC and LDL-C levels in healthy participants [115] and hypercholesterolemic patients [116], this recommendation severely limits an individual’s food choices. It also carries the risks of replacement by less healthful macronutrients, e.g., simple carbohydrates and low compliance, which might offset the benefit of SAFA restriction.

The short-term effects of dietary fatty acids on CVD risk are modest compared with drug treatment. However, dietary fats are ingested life-long by the entire population and have diverse effects on metabolism that may extend to next generations [117]. Therefore, the composition of dietary fat can have a large impact on public health. Wang et al. recently estimated that worldwide in 2010, a total of 711,800 CHD deaths could be attributed to low intake of n-6 PUFA, 250,900 CHD deaths to excess SAFA intake, and 537,200 CHD deaths to high TFA intake [39•]. Dietary fatty acids can also be an important adjunct to drug treatment [118].

Improving fatty acid intakes is an important element of diet and lifestyle approaches that can effectively lower the risk of CVD [28, 119, 120]. Recommendations for intakes of fatty acids and other nutrients are increasingly being translated into practical food-based dietary guidelines for the population. These unanimously advise limiting the consumption of SAFA-rich fats and foods, avoiding TFA, and consuming more foods rich in unsaturated fatty acids (Table 1). In practice, this implies the consumption of low-fat dairy products, lean meats, more vegetable oils, and vegetable oil-based foods, nuts, seeds, and the regular consumption of fatty fish. A healthy eating pattern also includes a greater intake of vegetables and fruits, carbohydrates from whole grains, a variety of protein foods (seafood, lean meat and poultry, eggs, legumes, and nuts), and low intakes of added sugar and sodium [106, 109].

## Conclusion

The type of dietary fat, but not total fat intake, is an important determinant of CVD risk. It is established by different types of studies that partial replacement of dietary SAFA with unsaturated fatty acids, in particular vegetable PUFA (mainly n-6 and some n-3) reduces the risk of CHD. Increasing intakes of PUFA are likely of greater benefit for cardiovascular health than further reducing SAFA intakes. Promising new studies indicate that n-6 PUFA may also reduce risk of T2DM, a major and modifiable risk factor for CVD. Although the clinical efficacy of supplemental n-3 VLCPUFA intake is not confirmed by recent RCTs, there is compelling evidence that regular consumption of fatty fish is related to lower risk of CHD.

Choosing the right types of food fats as part of a holistic diet and lifestyle approach to health can effectively reduce the risk of CVD and other chronic diseases, both in patients and in the general population.
